# Impact of Inter-Hospital Transfer on Outcomes in Patients Undergoing Emergency Abdominal Surgery: A Tertiary Referral Center’s Perspective

**DOI:** 10.1007/s00268-021-06174-5

**Published:** 2021-05-31

**Authors:** Joël L. Lavanchy, Jean-Baptiste Dubuis, Alice Osterwalder, Sebastian Winterhalder, Tobias Haltmeier, Daniel Candinas, Beat Schnüriger

**Affiliations:** Department of Visceral Surgery and Medicine, Inselspital, Bern University Hospital, University of Bern, 3010 Bern, Switzerland

## Abstract

**Background:**

In trauma patients, the impact of inter-hospital transfer has been widely studied. However, for patients undergoing emergency abdominal surgery (EAS), the effect of inter-hospital transfer on outcomes is largely unknown.

**Methods:**

This is a single-center, retrospective observational study. Outcomes of transferred patients undergoing EAS were compared to patients primarily admitted to a tertiary care hospital from 01/2016 to 12/2018 using univariable and multivariable analyses. The primary outcome was in-hospital mortality.

**Results:**

Some 973 patients with a median (IQR) age of 58.1 (39.4–72.2) years and a median body mass index of 25.8 (22.5–29.3) kg/m^2^ were included. The transfer group comprised 258 (26.3%) individuals and the non-transfer group 715 (72.7%). The population was stratified in three subgroups: (1) patients with low surgical stress (*n* = 483, 49.6%), (2) with hollow viscus perforation (*n* = 188, 19.3%) and (3) with potential bowel ischemia (*n* = 302, 31.1%). Neither in the low surgical stress nor in the hollow viscus perforation group was the transfer status associated with mortality. However, in the potential bowel ischemia group inter-hospital transfer was a predictor for mortality (OR 3.54, 95%CI 1.03–12.12, *p* = 0.045). Moreover, in the hollow viscus perforation group inter-hospital transfer was a predictor for reduced hospital length of stay (RC -10.02, 95%CI −18.14/−1.90, *p* = 0.016) and reduced severe complications (OR 0.38, 95%CI 0.18–0.77, *p* = 0.008).

**Conclusion:**

Other than in patients with low surgical stress or hollow viscus perforation, in patients with potential bowel ischemia inter-hospital transfer was an independent predictor for higher mortality. Taking into account the time sensitiveness of bowel ischemia, efforts should be made to avoid inter-hospital transfer in this vulnerable subgroup of patients.

## Introduction

The effect of inter-hospital transfer to tertiary referral centers on patient outcomes has been studied extensively in trauma patients [1–4] or patients suffering from ruptured aortic aneurysms [5, 6]. In non-trauma patients undergoing emergency abdominal surgery (EAS) for septic conditions, however, the literature on the effect of inter-hospital transfer is limited [7–10].

A delay to surgery has been associated with worse outcomes for emergency hernia surgery [11], appendectomy [12], cholecystectomy [13], large bowel perforation [14], small bowel obstruction [15, 16], acute mesenteric ischemia [17, 18] and for patients with surgical sepsis in general [19]. Not surprisingly, transferring a patient from hospital to hospital will increase the delay of these time-sensitive interventions [7]. However, it remains uncertain which group of patients is more vulnerable regarding inter-hospital transfer and in analogy to trauma-care which patients would profit the most from a regionalization of care.

Switzerland has a federal structure that leads to regionalized healthcare systems with small hospital catchment areas. To date, there are no established guidelines in Switzerland when to transfer EAS patients. Therefore, inter-hospital transfer of patients is subject to individual decision and resources of the sending hospital.

This study aims to assess the impact of inter-hospital transfer on multiple outcomes in three different subgroups of patients undergoing EAS. The subgroups are (1) patients with low surgical stress (appendicitis, cholecystitis) [20], (2) patients with hollow viscus perforation and (3) patients with potential bowel ischemia. We hypothesize that postoperative outcomes in transferred patients undergoing EAS for septic or time sensitive conditions are worse compared to non-transferred patients.

## Methods

### Study design

This is a single-center retrospective observational study. All patients undergoing non-trauma EAS at Bern University Hospital—an academic tertiary referral center—from January 2016 to December 2018 were included into the study. The Bern University Hospital has a catchment area of approximately one million inhabitants and is located in the northwestern part of Switzerland with a high-density of population. Overall, 14 regional hospitals are referring patients on a regular basis by ambulance or air transport. The maximum distance between referring hospitals and the study center is 100 km, which translates into a transfer time of 1 h by ambulance. Patients’ data were extracted from the institutional Acute Care Surgery database and electronic health records. Inclusion criteria were no objection to general consent, age ≥ 16 years, and one of the following EAS: appendectomy, cholecystectomy, laparoscopy/laparotomy for gastro-duodenal, small or large bowel perforation and anastomotic leakage, laparoscopy/laparotomy for incarcerated hernia, small or large bowel obstruction and acute mesenteric ischemia.

Patients referred from external hospitals were compared to patients directly admitted to the study center (transfer group vs. non-transfer group). Underlying pathologies were stratified in subgroup analysis based on the severity of illness and surgical management. Analysis was performed in three groups: (1) patients with low surgical stress (appendicitis, cholecystitis) [20], for (2) patients with hollow viscus perforation (gastro-duodenal, small bowel or large bowel and anastomotic leakage) and for (3) patients with potential bowel ischemia (incarcerated hernia, small or large bowel obstruction and mesenteric ischemia).

The primary outcome was in-hospital mortality. Secondary outcomes included intensive care unit (ICU) admission, ICU length of stay (ICU-LOS), length of mechanical ventilation, hospital length of stay (H-LOS) and complications graded according to the Dindo–Clavien classification [21].

### Statistical analysis

Categorical variables were reported as numbers and percentages and continuous variables as median and interquartile range (IQR). Normality of distribution was assessed using Shapiro–Wilk test. Categorical variables were compared using Fisher’s exact test and continuous variables using Mann–Whitney-U test. The effect of transfer on in-hospital mortality and secondary outcomes was adjusted using multivariable regression analysis. Patient characteristics (age, gender, body mass index (BMI) and quick sequential organ failure assessment (qSOFA) score [22] on admission) were assessed in univariable analysis and included into the multivariable model if the *p*-value was < 0.2. Linear or logistic regression analysis was used for continuous or binary outcomes, respectively. Results were reported as odds ratio (OR) or regression coefficients (RC) with 95% confidence intervals (CI). In sensitivity analysis, outcomes of patients with missing baseline characteristics were compared to outcomes of patients without missing baseline characteristics. *P-values* ≤ 0.05 were considered statistically significant. All statistical analyses were performed using SPSS Statistics version 25 (IBM Corporation, Armonk, New York).

### Ethical requirements

The cantonal ethics committee of Bern, Switzerland, approved the study protocol (KEK 2019-00,785). The study is reported in accordance with the STROBE (Strengthening the Reporting of Observational Studies in Epidemiology) statement [23].

## Results

### Patient characteristics

**Fig. 1:**
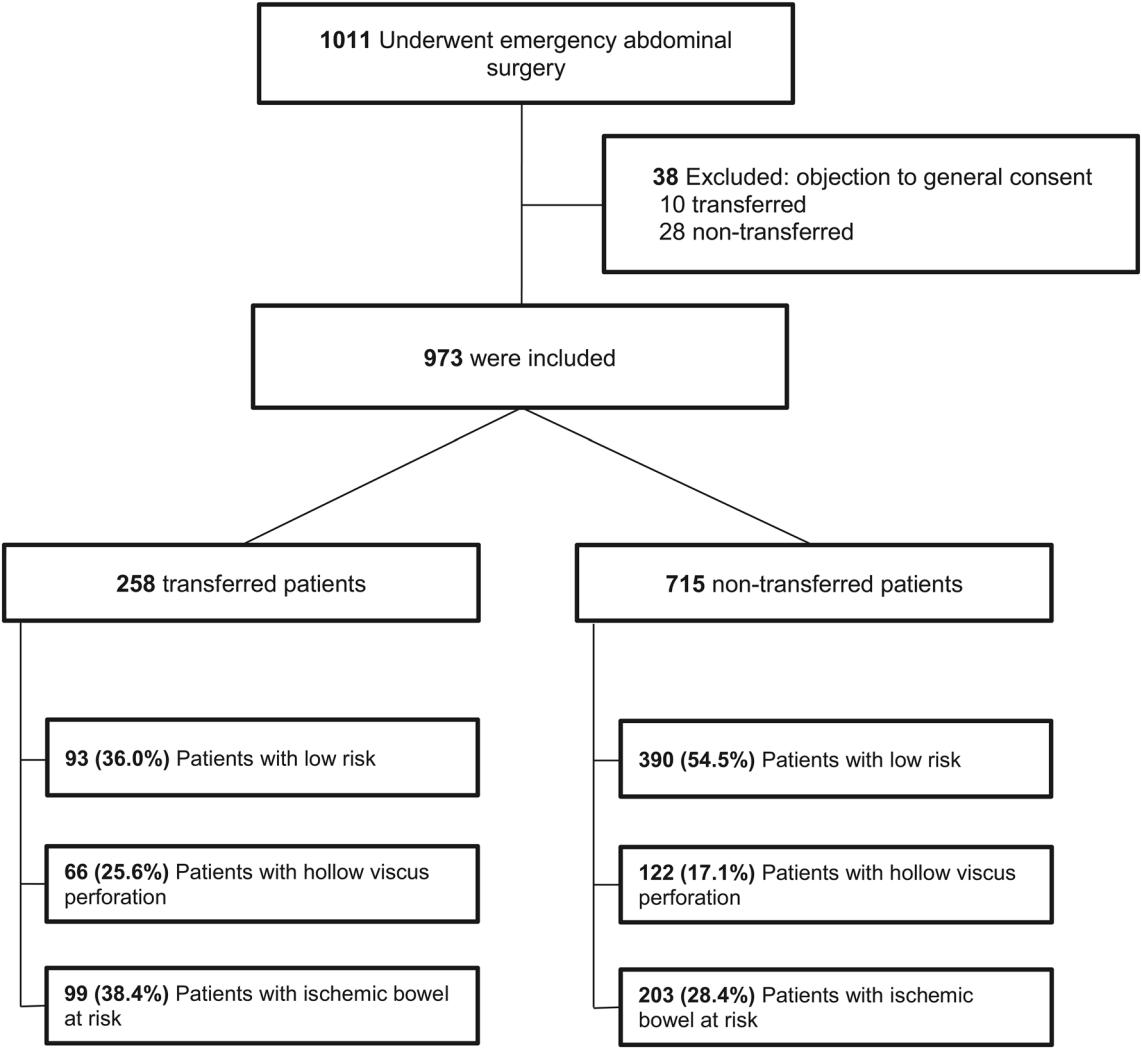
Flowchart of the study outline 215 × 279 mm (600 × 600 DPI)

During the 36-month study, 1011 patients underwent EAS with the above-mentioned inclusion criteria. Of these, 973 patients without objection to general consent were definitively included and further analyzed. Median (IQR) age was 58.1 (39.4–72.2) years and median BMI 25.8 (22.5–29.3) kg/m^2^ (Fig. 1). Of the study population, 26.5% (*n* = 258) were transferred from another hospital (transfer group) and 73.5% (*n* = 715) were directly admitted to the Bern University Hospital (non-transfer group). Baseline characteristics and indications for EAS are shown in Tables 1 and 2. The median age and BMI were significantly higher in the transfer group compared to the non-transfer group (66.5 vs. 55.0 years, *p* < 0.001; 26.2 vs. 25.5 kg/m^2^, *p* = 0.010). Moreover, the proportion of patients with a qSOFA score ≥ 2 was significantly higher in the transfer group compared to the non-transfer group (14.3% vs. 9.2%, *p* < 0.001). Sensitivity analysis revealed no differences in outcomes of patients with and without missing baseline characteristics.

**Table 1:** Baseline characteristics

	Total (*N* = 973)	Transfer (*n* = 258)	Non-transfer (*n* = 715)	*p*-value
Age, y, median (IQR)	58.1 (39.4–72.2)	66.5 (52.4–76.2)	55.0 (36.0–70.0)	** < 0.001**^**a**^
Sex, female (%)	447 (45.9)	117 (45.3)	330 (46.2)	0.827^b^
BMI, kg/m^2^, median (IQR)	25.8 (22.5–29.3)	26.2 (22.9–30.8)	25.5 (22.5–29.0)	**0.010**^**a**^
GCS, *n* (%)				
15	692 (71.1)	151 (58.5)	541 (75.7)	** < 0.001**^**b**^
< 15	73 (7.5)	30 (11.6)	43 (6.0)	
missing	208 (21.4)	77 (29.8)	131 (18.3)	
Systolic arterial blood pressure, *n* (%)				
> 100 mmHg	699 (71.8)	182 (70.5)	517 (72.3)	0.264^b^
≤ 100 mmHg	150 (15.4)	46 (17.8)	104 (14.5)	
missing	124 (12.7)	30 (11.6)	94 (13.1)	
Respiratory rate, *n* (%)				
≥ 22	294 (30.2)	113 (43.8)	181 (25.3)	** < 0.001**^**b**^
< 22	316 (32.5)	66 (25.6)	250 (35.0)	
missing	363 (37.3)	79 (30.6)	284 (39.7)	
qSOFA, *n* (%)				
≥ 2	103 (10.6)	37 (14.3)	66 (9.2)	**0.001**^**b**^
< 2	453 (46.6)	105 (40.7)	348 (48.7)	
Missing	417 (42.9)	116 (45.0)	301 (42.1)	

IQR: interquartile range; BMI: body mass index; GCS: Glasgow coma scale; qSOFA: quick sequential organ failure assessment; ^a^ Mann–Whitney *U* test, ^b^ Fisher’s exact test

Bold *p*-values are considered statistically significant

**Table 2:** Indications for emergency abdominal surgery

	Total (*N* = 973)	Transfer (*n* = 258)	Non-transfer (*n* = 715)
Low surgical stress (*n*, %)			
Appendicitis	274 (28.2)	38 (14.7)	236 (33.0)
Cholecystitis	209 (21.5)	55 (21.3)	154 (21.5)
Hollow viscus perforation (*n*, %)			
Large bowel perforation	70 (7.2)	22 (8.5)	48 (6.7)
Gastro-duodenal perforation	42 (4.3)	23 (8.9)	19 (2.7)
Small bowel perforation	39 (4.0)	13 (5.0)	26 (3.6)
Anastomotic leakage	37 (3.8)	8 (3.1)	29 (4.1)
Potential bowel ischemia (*n*, %)			
Small bowel obstruction	94 (9.7)	24 (9.3)	70 (9.8)
Incarcerated hernia	91 (9.4)	21 (8.1)	70 (9.8)
Mesenteric ischemia	64 (6.6)	35 (13.6)	29 (4.1)
Large bowel obstruction	53 (5.4)	19 (7.4)	34 (4.8)

### Outcomes of patients with low surgical stress

A total of 483 patients underwent appendectomy or cholecystectomy and were defined as patients with low surgical stress: 93 patients (19.3%) in the transfer group and 390 patients (80.7%) in the non-transfer group. Median age and BMI were significantly higher in the transfer group compared to the non-transfer group (66.5 vs. 43.0 years, *p* < 0.001; 27.0 vs. 25.8 kg/m^2^, *p* = 0.019). Moreover, qSOFA scores ≥ 2 were significantly more frequent in the transfer vs. the non-transfer group (29.4% vs. 10.6%, *p* < 0.001).

There was a trend toward more complication in transferred patients (16.1% vs. 8.7%, *p* = 0.054). Moreover, ICU admission rate was significantly increased in the transfer group compared to the non-transfer group (9.7% vs. 3.8%, *p* = 0.031). Additionally, median ICU-LOS and H-LOS were significantly longer in the transfer group compared to the non-transfer group (3.0 vs. 1.7 days, *p* = 0.042; 4.0 vs. 3.0 days, *p* < 0.001).

In multivariable analysis, inter-hospital transfer was not associated with worse outcomes in this subgroup (*Table **3*). ICU admission was independently predicted by increased age (OR 1.04, 95%CI 1.01–1.07, *p* = 0.031) and qSOFA scores ≥ 2 (OR 7.07, 95%CI 2.38–20.96, *p* < 0.001). Furthermore, higher BMI was independently associated with more ventilator days (RC 0.02, 95%CI 0.09–0.34, *p* = 0.012), higher age with longer H-LOS (RC 0.06, 95% CI 0.04–0.09, *p* < 0.001) and qSOFA scores ≥ 2 with longer H-LOS (RC 2.83, 95%CI 1.34–4.32, *p* < 0.001)*.*

**Table 3:** Effect of baseline characteristics on outcomes in patients with low surgical stress (appendicitis, cholecystitis), *N* = 483

	Univariable		Multivariable	
	OR/RC (95% CI)	*p*-value	OR/RC (95% CI)	*p*-value
ICU admission				
Transfer	2.68 (1.13–6.32)	**0.031**	2.76 (0.87–8.71)	0.084
Age	1.05 (1.03–1.08)	** < 0.001**	1.04 (1.01–1.07)	**0.031**
BMI	1.09 (1.02–1.17)	**0.017**	1.05 (0.94–1.17)	0.382
qSOFA ≥ 2	11.38 (4.02–32.20)	** < 0.001**	7.07 (2.38–20.96)	** < 0.001**
ICU-LOS				
Transfer	2.36 (-0.18–4.91)	0.067	1.74 (-0.75–4.23)	0.160
Age	0.04 (-0.01–0.09)	0.084	0.02 (-0.03–0.07)	0.319
BMI	0.21 (0.02–0.41)	**0.035**	0.17 (-0.02–0.37)	0.074
Ventilation days				
Transfer	1.30 (-1.88–4.48)	0.354	0.71 (-0.89–2.32)	0.251
BMI	0.23 (0.09–0.37)	**0.007**	0.21 (0.09–0.34)	**0.012**
qSOFA ≥ 2	1.95 (-1.93–5.82)	0.254	1.44 (-0.37–3.25)	0.085
H-LOS				
Transfer	1.99 (0.96–3.01)	** < 0.001**	0.52 (-0.81–1.86)	0.441
Age	0.08 (0.06–0.10)	** < 0.001**	0.06 (0.04–0.09)	** < 0.001**
qSOFA ≥ 2	3.84 (2.35–5.33)	** < 0.001**	2.83 (1.34–4.32)	** < 0.001**
Complications ≥ 3a				
Transfer	2.00 (0.74–5.41)	0.229	1.61 (0.56–4.65)	0.375
Age	1.02 (1.00–1.04)	0.127	1.01 (0.99–1.04)	0.241
Mortality				
Transfer	8.55 (0.77–95.21)	0.096	5.57 (0.35–88.40)	0.223
Age	1.04 (0.98–1.11)	0.172	1.03 (0.97–1.10)	0.352
BMI	0.76 (0.53–1.09)	0.139	0.75 (0.53–1.05)	0.097

OR: odds ratio; RC: regression coefficient; CI: confidence interval; BMI: body mass index; qSOFA: quick sequential organ failure assessment; IQR: interquartile range; LOS: length of stay; ICU: intensive care; H-LOS: hospital length of stay

Bold *p*-values are considered statistically significant

### Outcomes of patients with hollow viscus perforation

Overall, 188 patients with hollow viscus perforation were included into the study. Thereof, 66 patients (35.1%) were in the transfer group and 122 patients (64.9%) in the non-transfer group. Hollow viscus perforations were located in the large bowel in 37.2% (*n* = 70), gastro-duodenal in 22.3% (*n* = 42), in the small bowel in 20.7% (*n* = 39) and due to anastomotic leakage in 19.7% (*n* = 37) of the patients. There were significantly more patients with gastro-duodenal perforations in the transfer group compared to the non-transfer group (8.9% vs. 2.7%, *p* < 0.001). The median age, BMI and qSOFA score were not statistically different in the transfer and the non-transfer group*s.*

The mortality of patients with hollow viscus perforation was comparable in the transfer and the non-transfer groups (11.5% vs. 9.1%, *p* = 0.743). However, complications ≥ 3a were significantly less frequent and median H-LOS was significantly lower in the transferred compared to the non-transferred population (22.7% vs. 41.8%, *p* = 0.010; 11.0 vs. 16.0 days, *p* = 0.003).

Multivariable analysis revealed age as an independent predictor for ICU admission (OR 1.05, 95%CI 1.02–1.08, *p* = 0.001). Moreover, inter-hospital transfer reduced H-LOS (RC -10.02, 95%CI −18.14/−1.90, *p* = 0.016) and complications ≥ 3a (OR 0.38, 95%CI 0.18–0.77, *p* = 0.008) significantly (Table 4).

**Table 4:** Effect of baseline characteristics on outcomes in patients with hollow viscus perforation (gastro-duodenal, small bowel or large bowel perforation, anastomotic leakage), *N* = 188

	Univariable		Multivariable	
	OR/RC (95% CI)	*p*-value	OR/RC (95% CI)	*p*-value
ICU admission				
Transfer	1.11 (0.61–2.02)	0.735	1.39 (0.56–3.45)	0.482
Age	1.05 (1.03–1.07)	** < 0.001**	1.05 (1.02–1.08)	**0.001**
qSOFA ≥ 2	2.06 (0.91–4.65)	0.083	1.87 (0.78–4.45)	0.158
ICU-LOS				
Transfer	−1.20 (−5.30–2.89)	0.561	−1.04 (−6.88–4.79)	0.720
qSOFA ≥ 2	4.89 (−0.92–10.70)	0.097	4.86 (−1.03–10.74)	0.103
Ventilation days				
Transfer	0.83 (−2.78–4.44)	0.648	1.15 (−2.40–4.71)	0.520
Female gender	−3.22 (−6.60–0.16)	0.061	−3.32 (−6.73–0.09)	0.056
H-LOS				
Transfer	−10.14 (−18.28 to −2.00)	**0.015**	−10.02 (−18.14 to −1.90)	**0.016**
Age	0.18 (−0.06–0.43)	0.146	0.18 (−0.07–0.42)	0.155
Complications ≥ 3a				
Transfer	0.41 (0.21–0.81)	**0.010**	0.38 (0.18–0.77)	**0.008**
Age	1.02 (1.00–1.04)	**0.035**	1.02 (1.00–1.04)	0.064
BMI	1.04 (0.98–1.09)	0.183	1.04 (0.99–1.10)	0.119
Female gender	1.63 (0.89–2.97)	0.114	1.38 (0.72–2.66)	0.337
Mortality				
Transfer	0.77 (0.28–2.11)	0.614	1.11 (0.26–4.87)	0.886
Age	1.05 (1.01–1.09)	**0.012**	1.05 (1.00–1.11)	0.057
Female gender	2.48 (0.94–6.52)	0.066	0.74 (0.19–2.88)	0.661
qSOFA ≥ 2	4.04 (1.10–14.89)	**0.036**	3.85 (0.99–14.95)	0.052

OR: odds ratio; RC: regression coefficient; CI: confidence interval; BMI: body mass index; qSOFA: quick sequential organ failure assessment; IQR: interquartile range; LOS: length of stay; ICU: intensive care; H-LOS: hospital length of stay

Bold *p*-values are considered statistically significant

### Outcomes of patients with potential bowel ischemia

A total of 302 patients with preoperatively suspected bowel ischemia (incarcerated abdominal wall hernia, small or large bowel obstruction and mesenteric ischemia) were included into the study. Thereof, 99 patients (32.8%) were in the transfer group and 203 (67.2%) in the non-transfer group. There were significantly more patients with mesenteric ischemia in the transfer group compared to the non-transfer group (13.6% vs. 4.1%, *p* < 0.001). All other diagnoses (small bowel obstruction, incarcerated hernia and large bowel obstruction) were comparable between the two groups (Table 2). The median BMI was significantly higher in the transfer group compared to the non-transfer group (26.0 vs. 24.7 kg/m^2^, *p* = 0.044). The median age and qSOFA scores ≥ 2 were not significantly different between the transfer and the non-transfer groups.

Mortality of patients with potential bowel ischemia was significantly higher in the transfer group compared to the non-transfer group (16.2% vs. 4.4%, *p* < 0.001). Moreover, ICU admission rates were significantly increased in the transferred compared to the non-transferred population (38.4% vs. 26.1% *p* = 0.033).

Multivariable regression analysis revealed age (OR 1.04, 95%CI 1.02–1.07, *p* = 0.002) and qSOFA scores ≥ 2 (OR 6.54, 95%CI 2.56–16.70, *p* < 0.001) as independent predictors for ICU admission. Furthermore, qSOFA scores ≥ 2 were independently associated with more ventilator days (RC 5.12, 95%CI 1.13–9.11, *p* = 0.013) and H-LOS (RC 6.36, 95%CI 1.24–11.48, *p* = 0.015). Inter-hospital transfer (OR 3.54, 95%CI 1.03–12.12, *p* = 0.045), increased age (OR 1.08 95%CI 1.02–1.14, *p* = 0.008) and qSOFA scores ≥ 2 (OR 6.95 95%CI 1.96–24.64, *p* = 0.003) predicted in-hospital mortality (Table 5).

**Table 5:** Effect of transfer status on outcomes of patients with potential bowel ischemia (incarcerated hernia, small and large bowel obstruction, mesenteric ischemia), *N* = 302

	Univariable		Multivariable	
	OR/RC (95% CI)	*p*-value	OR/RC (95% CI)	*p*-value
*ICU admission*				
Transfer	1.76 (1.06–2.94)	**0.030**	1.68 (0.82–3.44)	0.159
Age	1.04 (1.02–1.06)	** < 0.001**	1.04 (1.02–1.07)	**0.002**
qSOFA ≥ 2	6.73 (2.74–16.53)	** < 0.001**	6.54 (2.56–16.70)	** < 0.001**
*ICU-LOS*				
Transfer	0.40 (−2.01 to 2.81)	0.741	− 0.05 (−2.72 to 2.61)	0.969
qSOFA ≥ 2	7.27 (3.93–10.61)	** < 0.001**	7.27 (3.89–10.65)	** < 0.001**
*Ventilation days*				
Transfer	− 0.34 (−2.86 to 2.17)	0.787	− 0.66 (−3.80 to 2.48)	0.672
qSOFA ≥ 2	5.05 (1.12–8.98)	**0.013**	5.12 (1.13–9.11)	**0.013**
*H-LOS*				
Transfer	− 0.17 (−3.24 to 2.91)	0.915	− 0.99 (−5.04 to 3.06)	0.631
Age	0.10 (0.02–0.19)	**0.018**	0.09 (−0.02 to 0.20)	0.103
qSOFA ≥ 2	6.63 (1.54–11.73)	**0.011**	6.36 (1.24–11.48)	**0.015**
*Complications ≥ 3a*				
Transfer	1.89 (1.07–3.34)	**0.028**	1.64 (0.77–3.51)	0.202
Age	1.02 (1.01–1.04)	**0.012**	1.01 (0.98–1.03)	0.692
qSOFA ≥ 2	3.40 (1.44–8.05)	**0.005**	3.23 (1.35–7.71)	**0.008**
*Mortality*				
Transfer	4.16 (1.77–9.78)	**0.001**	3.54 (1.03–12.12)	**0.045**
Age	1.07 (1.04–1.11)	** < 0.001**	1.08 (1.02–1.14)	**0.008**
qSOFA ≥ 2	6.52 (2.08–20.48)	**0.001**	6.95 (1.96–24.64)	**0.003**

OR: odds ratio; RC: regression coefficient; CI: confidence interval; BMI: body mass index; qSOFA: quick sequential organ failure assessment; IQR: interquartile range; LOS: length of stay; ICU: intensive care; H-LOS: hospital length of stay

Bold *p*-values are considered statistically significant

## Discussion

In the current study, the impact of a preoperative inter-hospital transfer on outcomes in 983 patients undergoing EAS was assessed. Patients transferred for EAS to a tertiary hospital were significantly older and had a higher BMI compared to patients directly admitted. In addition, patients in the transfer group were significantly more often in a septic condition based on qSOFA scores ≥ 2 on admission. In the subgroup of patients with potential bowel ischemia, the transfer status was independently associated with increased mortality.

To our knowledge, there is only one previous single-center study looking at inter-hospital transfer in patients undergoing EAS. In line with the current analysis, in this study inter-hospital transfer was associated with significantly more comorbidities, higher mortality and longer H-LOS [9]. However, compared to the current study, the study population was smaller and no subgroup analysis was performed. In the current study, in order to reduce heterogeneity, analysis was performed in three separate groups [(1) low surgical stress, (2) hollow viscus perforation and (3) potential bowel ischemia].

In patients with *low surgical stress*, transfer status had no impact on mortality; however, transferred patients presented with older age, higher BMI and higher qSOFA scores. This resulted in a higher ICU admission rate and longer H-LOS. After adjustment in multivariable regression analysis, age and higher qSOFA scores, but not transfer status remained as independent predictors for ICU admission and longer H-LOS. In this context, the transfer status may be seen as a surrogate for patients with increased perioperative risks, including older age, higher BMI and higher qSOFA scores. However, clarification of cause and effect of this observation needs further investigations.

Interestingly, in patients with *hollow viscus perforation*, transfer status had no significant impact on ICU admission and mortality. Moreover, in this subgroup, patients’ characteristics, including qSOFA scores, were comparable between the transferred and non-transferred groups. It has been shown that patients with septic abdominal conditions benefit from a preoperative course of i.v. fluids and antibiotic treatment before surgical source control [24]. In this group of patients, a delay to surgery might be less important. Moreover, in this subgroup of patients, the transfer group had a shorter H-LOS and less complications ≥ 3a compared to non-transferred patients. This finding may be explained by a liberal re-transfer policy once source control is achieved and patients are hemodynamically stable.

In patients with *potential bowel ischemia*, the transfer status had a strong impact on mortality even after multivariable adjustment. This finding underlines the time sensitiveness of surgical intervention in patients suffering from bowel ischemia. Transferring a patient from hospital to hospital will increase the delay to definitive care [7]. Based on the findings of the current study, in patients with bowel ischemia efforts should be made to shorten the prehospital time including avoidance of inter-hospital transfer.

The findings of the current study are limited by its retrospective nature. Moreover, the respiration rate was not routinely documented and qSOFA score infrequently calculated at admission. However, sensitivity analysis did not reveal significant differences in outcomes between patients with and without documented qSOFA score at admission. Furthermore, this study is limited to the referral center’s perspective of inter-hospital transfer.

In summary, transfer status may be seen as a surrogate marker for higher perioperative risks in patients undergoing EAS that translates into worse outcomes. The subgroup of transferred patients with potential bowel ischemia or patients with increased comorbidities such as older age [25, 26] is a vulnerable patient population that need special attention with extensive treatment needs. Direct admission to a tertiary care center in order to avoid inter-hospital transfer is preferable in this vulnerable patient population. Whether a categorization of hospitals as implemented in trauma care [27, 28] would improve outcomes in patients requiring EAS for non-trauma disease needs further careful assessment [29]. Of note, in order to balance the benefits vs. risks of inter-hospital transfers, it is of paramount importance to stratify the patients according the underlying acute disease. One possible differentiation of patients requiring EAS for non-trauma disease is suggested in the current study.

### Conclusion

Other than in patients with low surgical stress or hollow viscus perforation, in patients with potential bowel ischemia inter-hospital transfer was an independent predictor for higher mortality. Taking into account the time sensitiveness of bowel ischemia, efforts should be made to shorten the prehospital time including avoidance of inter-hospital transfer in this vulnerable subgroup of patients. Whether a categorization of hospitals regarding the level of care in EAS for non-trauma disease would improve outcomes needs further careful assessment.
